# Pilot introduction of long-lasting insecticidal nets and hammock nets in the indigenous Comarca of Guna Yala, Panama

**DOI:** 10.1186/s12936-024-05208-2

**Published:** 2024-12-18

**Authors:** A. Oscar E. González, Carmen Perez, Tania Blanco, Cipriano Ayarza, Santiago Chérigo, Mario Ávila, Lucía Fernández Montoya, Nicholas A. Presley, Bernardo García Espinosa, Mariela Mosquera Renteria

**Affiliations:** 1 Departamento de Control de Vectores, Ministry of Health of the Republic of Panama, Panama City, Panama; 2 Clinton Health Access Initiative (CHAI), Boston, USA

**Keywords:** Malaria, Long-lasting insecticidal nets (LLIN), Long-lasting insecticidal hammock nets (LLIHN), Indigenous populations

## Abstract

**Background:**

After almost 70 years of using indoor residual spraying (IRS) as the primary intervention for malaria vector control, the Republic of Panama wanted to evaluate the operational feasibility and acceptability of distributing long-lasting insecticidal hammock nets (LLIHNs) and long-lasting insecticidal nets (LLINs) in the country.

**Methods:**

A pilot study conducted in 2019 distributed LLINs and LLIHNs to cover all sleeping spaces in 15 high burden localities of the indigenous Comarca of Guna Yala and measured retention, coverage, use and physical deterioration, washing and drying practices, as well as people’s satisfaction with product characteristics post-distribution.

**Results:**

Overall, 89.9% of enumerated sleeping spaces were covered during the campaign. Monitoring post-distribution showed that 82.7% of the population received messages about the campaign before it happened and 92.4% claimed to know the purpose of the net and how to care for and repair it. Mild adverse reactions, specifically skin irritation associated with the insecticide in LLINs and LLIHNs, were reported by 38.4% of households. Two years after distribution, 86.3% of the LLIHN/LLINs were retained. Use was very high right after distribution (85%) but decreased to 57% six months after distribution and to 38% two years after distribution. The main reason for not using the LLIHN/LLINs was the reported absence of mosquitoes. Two years post-distribution, LLIHN/LLINs were preserved in good physical condition (4% torn), very few were washed with insecticide-damaging products (chlorine or detergent) (9%) or dried under the sun (15%), and LLIHN/LLINs were washed on average less than once every two months. The average number of people per sleeping space was 1.34.

**Conclusion:**

Although the distribution of LLIHN/LLINs was operationally feasible and LLIHN/LLINs were initially well-accepted and cared for by these communities, use decreased drastically over the two years of follow up after distribution. Hence, should there be future LLIHN/LLIN distributions in this area, sufficient resources and efforts need to be allocated to promoting LLIHN/LLIN use. Further investigation into the reasons for low LLIHN/LLIN use are needed to guide such efforts.

**Supplementary Information:**

The online version contains supplementary material available at 10.1186/s12936-024-05208-2.

## Background

Malaria is endemic in Panama. Most malaria cases are caused by *Plasmodium vivax* and 99% of malaria cases are concentrated in mostly indigenous villages scattered throughout four regions of the country: Guna Yala, Panama Este, Darién, Ngäbe-Buglé [1]. Based upon a consistent decrease in cases over the previous eight years, in 2018, the Republic of Panama developed the first national plan for malaria elimination, committing to eliminate malaria and certify elimination by 2025 [2]. One year later, Panama joined the Regional Malaria Elimination Initiative (IREM) in Central America [3].

In Panama, indoor residual spraying (IRS) DDT, organophosphates, pyrethroids and neonicotinoids has been the main vector control intervention since the 1940s. However, in 2018, acknowledging the need to use several interventions to achieve effective control of malaria vectors, the Ministry of Health adopted an integrated vector management approach and set the goal to achieve full coverage with either long-lasting insecticidal nets (LLINs) or IRS in target communities across all active transmission foci, as well as to use other complementary interventions, such as larvicides or environmental management, in high-risk areas [4].

Panama had previously piloted long-lasting insecticidal bed nets (LLINs) in 2008, but with limited impact, due to LLINs being distributed in malarious regions where a high proportion of the population slept in hammocks rather than beds/sleeping pads [2]. Based on this experience, Panama was interested in introducing long-lasting insecticidal hammock nets (LLIHN) alongside LLINs. The country was further encouraged by the impact of LLIHNs seen in other countries in Latin America [5] and by the fact that LLINs were becoming the main vector control intervention in most countries [6]. In Indigenous areas of the Venezuelan Amazon, 56% of malaria cases were prevented two years after the distribution of manually insecticide-treated hammock nets [5]. In Suriname, a reduction in malaria incidence was observed between 1989 and 1991 following the distribution of treated hammock nets [7].

In 2018, the Panamanian Ministry of Health decided to evaluate the operational feasibility and acceptability of LLIHN and LLIN distributions. A pilot study was designed to distribute sufficient LLIHNs and LLINs to cover all sleeping spaces in 15 localities in the Indigenous Comarca of Guna Yala and to measure LLIHN/LLIN retention, use, physical deterioration, washing and drying practices, as well as people’s satisfaction with LLIHN/LLIN characteristics after distribution. This manuscript reports the results of the pilot study which was used to inform the implementation of a subsequent national LLIHN/LLIN campaign in 2023.

## Methods

### Study site

The Guna Yala Comarca is a semi-autonomous indigenous territory within the Caribbean coastline of the Republic of Panama. It shares a border with the Republic of Colombia to the east. The estimated 46,267 residents registered in 2019 [8] were primarily of the Guna indigenous group, although two communities (La Miel and Puerto Obaldia) located near the Colombian border are ethnically Afro-Panamanian. Each Guna community is led by a *saila* and decisions are made in community meetings regularly held at *Casas de Congreso*. Guna people communicate in their language and a high percentage of people sleep in hammocks [9]. The main malaria vector is *Anopheles albimanus*, whose density peaks during the rainy season, who bites more outdoor than indoor and whose peak of biting occurs during the early evening [10].

**Fig. 1:**
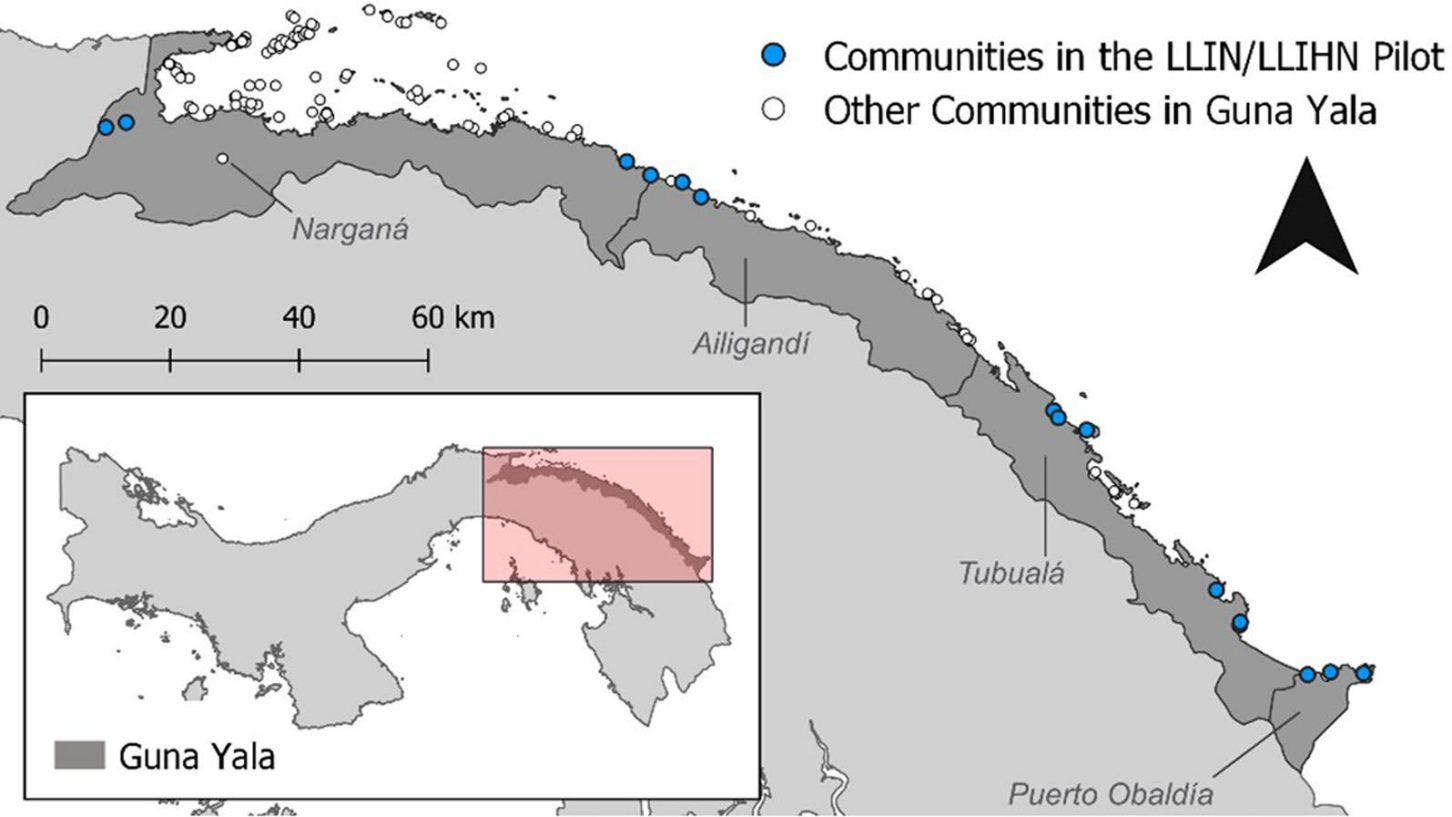
Map of Guna Yala showing the localities that received LLHIN/LLIN during the pilot household was collected through a paper form (Additional file 1 form 2)

The Ministry of Health (MoH) chose to conduct the LLHIN/LLIN pilot in the Guna Yala region due to its history of high malaria endemicity and unique vulnerabilities. In 2018, nearly 40% of the cases nationwide were detected in Guna Yala [11]. The selection of communities for this pilot was based on an analysis of historical disease burden, where 13 localities were identified to report an incidence of over 5 cases per 1000 inhabitants between 2014 and 2017 (Fig. 1). Additionally, two Afro-Panamanian communities were included because of their proximity to these high-risk malaria areas, as well as their distinct environmental and social characteristics.

### Household enumeration and LLIN quantification

To determine the number of nets to be procured and set targets for the distribution, an enumeration of households and sleeping spaces was conducted between September and October 2017, counting all beds, hammocks, and other sleeping spaces (see Additional file 1 Form 1). A buffer of 10% was applied over the number of beds and hammocks censed to account for any households not enumerated or population changes between the enumeration and the distribution date.

### Social Behaviour Change Communication (SBCC)

SBCC activities were conducted before, during and after the campaign, targeting children, women, men, *sailas*, community health workers (CHW), local vector control technicians and school teachers to foster community acceptance and use of LLIHN/LLINs. Before the campaign, messages were disseminated via local Guna radio, and posters were hung in the offices of vector control technicians, at health centers, migration control points, stores, the houses of CHWs, and in the house of the Guna Congress in each locality. During the distribution, demonstrations of how to use LLIHN/LLINs were provided during community meetings (Congreso), and oral instructions and a leaflet were given to each household. After the distribution, CHWs continued disseminating information about net use and handling, and posters were hung at schools. Such post-campaign efforts were concentrated in the first-year post-distribution.

### Net distribution

**Fig. 2:**
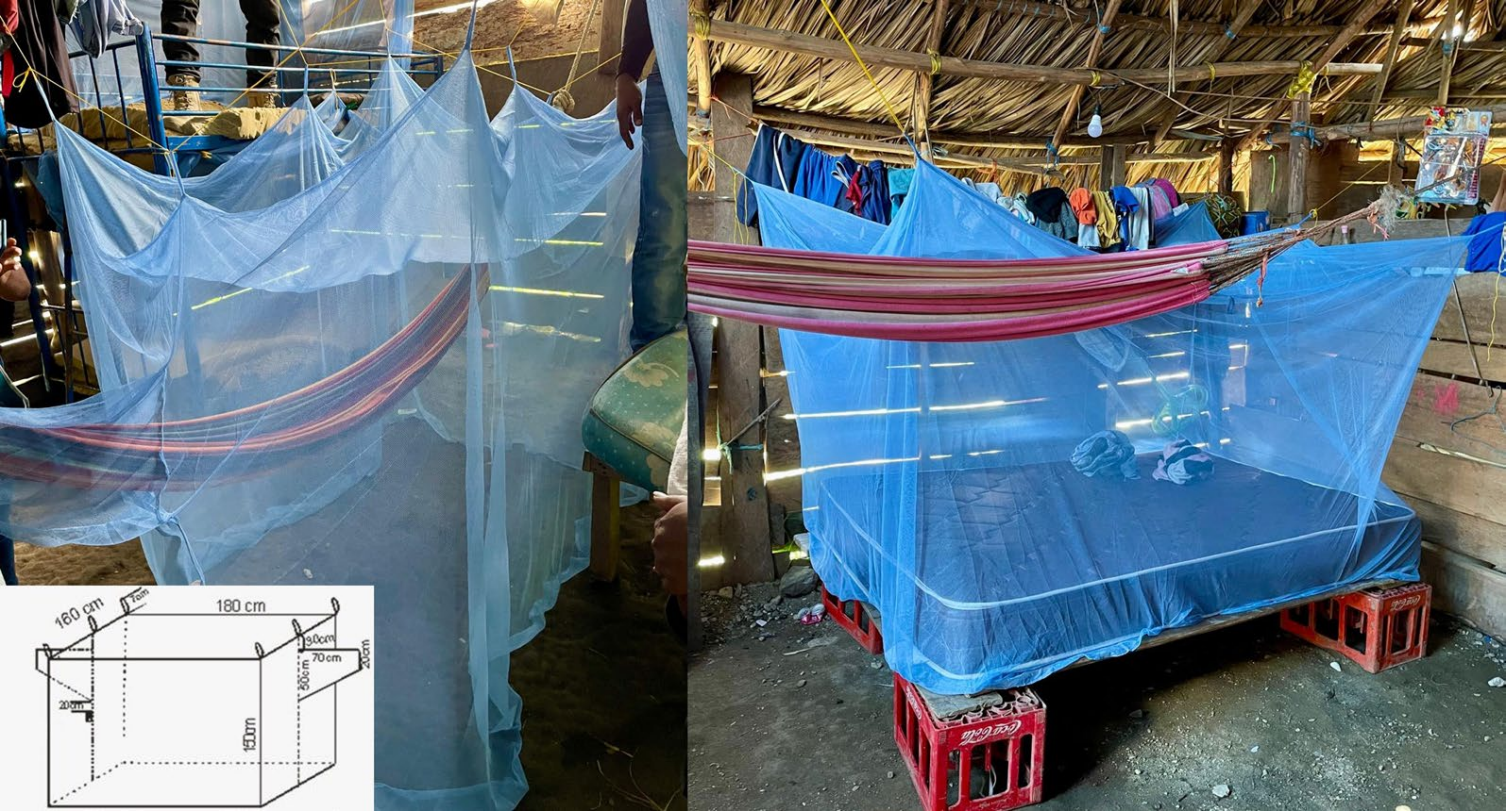
Long-lasting insecticidal hammock nets (LLIHNs, left image) and long-lasting insecticidal bed nets (LLINs, right image)

Permanet 2.0 (Vestergaard Sarl) bed nets (LLINs) and hammock nets (LLIHNs) (Fig. 2), made of polyester coated with deltamethrin at a concentration of 1.4 g/kg, were distributed and installed door-to-door from June to August 2019 by vector control technicians and trained community volunteers. To avoid adverse reactions due to exposure to recently opened nets, all nets were removed from their plastic packaging and hung in an empty house on clotheslines 24 h before distribution. At the end, a mop-up round was conducted to distribute nets to households that were closed during the main distribution.

### Post-distribution verification

Two days after completing net distribution in each locality, post-distribution verification visits were conducted to verify whether the households had received LLIHN/LLINs for all sleeping spaces and to determine net usage immediately after distribution (Additional file 1—form 3). The aim was to visit all households in the targeted localities. The verification was done by the supervisors of the distribution teams by visiting these households and visually verifying all LLIHN/LLINs installed during the campaign over the sleeping spaces.

### Post-distribution monitoring and evaluation

After distribution, two cross-sectional surveys were conducted to monitor LLIHN/LLIN retention, coverage, use, physical integrity, washing and drying practices, and population satisfaction with the nets. The first took place in February 2020 (dry season), six months after the distribution, and the second from July to November 2021 (rainy season), two years after the distribution. The aim was to visit all households in the localities that received LLINs/LLIHNs. Net presence was visually verified by the survey teams. Physical integrity was measured in the field as per World Health Organization (WHO) Guidelines [12] in 4 LLIHN/LLINs per household (or less if the household had less LLIHN/LLINs). After the first round of monitoring, several questions were added to evaluate the outreach of the SBCC efforts made before and during the campaign. The full questionnaires used in the two rounds of monitoring are available in Additional file 1—Form 4 and 5.

### Data collection

Data from enumeration, distribution, verification and the first post-distribution monitoring survey were collected on paper by trained Vector Control Technicians. Filled forms were individually reviewed by staff from the National Malaria Control Programme (NMCP) and Clinton Health Access Initiative (CHAI) to ensure data consistency and were later entered into tailored Epi Info data collection screens by trained data entry staff. Owing to the adoption of District Health Information System − 2 (DHIS2) as the common platform for data collection and visualization by the Vector Control Department, data for the second post-distribution monitoring survey were collected on DHIS2 version 2.36 [13] using a DHIS2 Tracker program specially designed for the campaign.

#### Data analysis

Data were analysed with R version 4.3.1 [14] and graphs were prepared using the package ggplot2 [15]. Table 1 in Additional File 2 lists and defines all the indicators reported from the distribution, verification, and monitoring phases of the project, with their numerator and denominator indicated. Most indicators, including coverage, use, and retention, were calculated as proportions of households or sleeping spaces that met specific conditions (see Additional file 2). The only indicator quantified by a mean was the number of times that nets were washed in the last 6 months. The Proportional Hole Index (pHI) was calculated for each evaluated LLIHN/LLIN following WHO guidelines [12] and was used to calculate the percentage of LLIHN/LLINs in good (pHI < 64), damaged (pHI 65–642) and torn physical conditions (pHI ≥ 643). Where possible, values are disaggregated by type of net (LLIHN or LLIN), by locality and by monitoring round. Two proportion Z-tests, t-tests and Fisher’s Exact tests were run to evaluate differences between monitoring rounds and between LLIHNs and LLINs.

## Results

### Distribution results

A total of 4818 sleeping spaces were registered during the 2017 enumeration, 65% (*n* = 3140) were hammocks and 35% (*n* = 1678) were beds, leading to the procurement of 3430 LLHNs and 1830 LLINs. The campaign distributed LLIHN/LLINs across 925 households. During the campaign, a total of 4433 sleeping spaces were registered, 2765 hammocks and 1668 beds. In 11 out of the 15 localities, fewer sleeping spaces were registered during the campaign than during the 2017 enumeration. A total of 2723 LLIHNs and 1609 LLINs were installed (Additional File 3—Table 1).

**Fig. 3:**
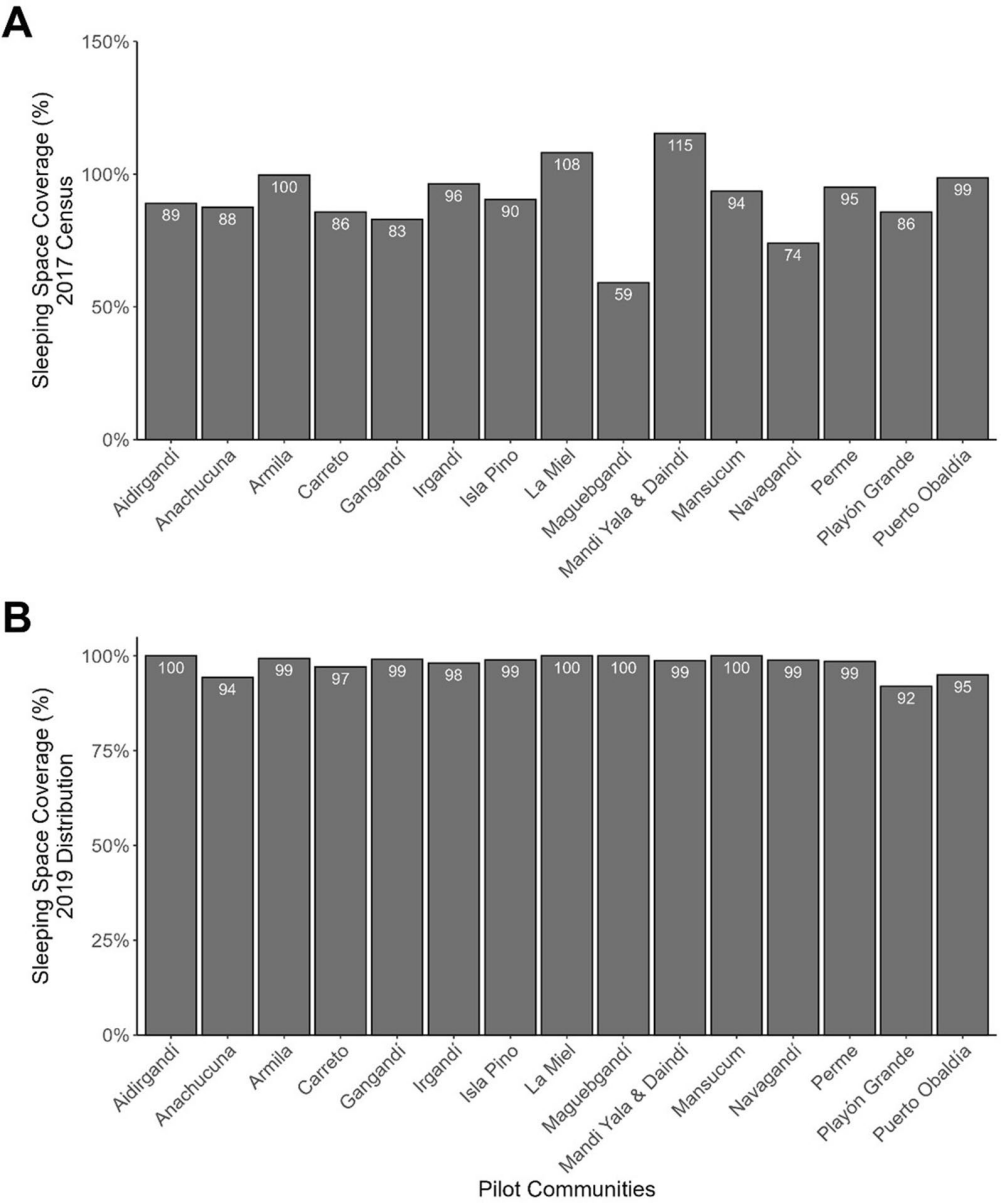
Percentage of sleeping spaces covered with an LLIHN or an LLIN by the end of distribution per **A** spaces from the 2017 census and **B** spaces quantified during distribution

Based on the enumeration data of 2017, the campaign covered 89.9% of enumerated sleeping spaces with an LLIHN/LLINs (86.7% of hammocks and 95.9% of beds), with two of the 15 communities reaching coverage of over 100% (Fig. 3A). Of the sleeping spaces found during distribution, 97.7% were covered with an LLIHN or an LLIN (98.5% of the hammocks and 96.5% of the beds) (Fig. 3B). Overall, 4.2% of the households required a second visit to complete the distribution. At the time of distribution, 59.1% of the households already had some type of untreated net, anecdotally noted to be either made of fine mesh polyester or crafted by people using local fabric. In households that were reached during the campaign, the average number of people per household was 5.71 and the average number of people per sleeping space was 1.34.

During verification post-distribution, not all households were visited due to lack of field personnel, but a total of 423 households were indeed visited. It was observed that 95.6% of them had received at least one net per sleeping space during the distribution.

### Social Behaviour Change Communication (SBCC)

At the time of distribution, 82.7% of the households reported that they had previously received messages about the upcoming distribution through radio spots or brochures. During verification, 94.4% of the households claimed to have received information from the distribution team, 96.6% claimed to know the purpose of the net, 96.1% claimed to know how to wash it, 96.6% how to dry it, 95.9% how to handle the net when not in use and 95.6% how to repair holes that might appear. Overall, 92.4% of households where verification occurred gave positive or appropriate answers to all LLIHN/LLIN management questions.

### Post-distribution monitoring results

A total of 548 households were visited in the first monitoring round and 463 in the second. The difference is due to the fact that two localities were excluded from the second monitoring round (Carreto and Navagandi) due to lack of available personnel to conduct the fieldwork.

### Satisfaction with the distributed nets

Six months after distribution, 93.3% of respondents claimed being satisfied with their LLIHN/LLINs’ colour (blue), 93.2% with the fabric (Polyester), 94.9% were satisfied with the size of their LLINs (190 × 180 × 150 cm) and 92.1% with the size of their LLIHNs (160*180*150 cm). Two years after distribution, these numbers dropped to 76.5% satisfaction with LLIN color, 73.5% satisfied with their fabric, and 78.0% with LLIN size. Satisfaction with LLIHN size changed the least, with 86.8% of respondents still finding their size agreeable. Overall, 83.9% of surveyed households claimed to be satisfied with all LLIN characteristics (colour, fabric, and size) six months after distribution and 48.2% two years after distribution (z-test p < 0.001).

### Adverse reactions

Six months after distribution, mild adverse reactions to LLIHN/LLINs were reported across 38.4% of the households where monitoring occurred, with 17.1% of the people in surveyed households claiming to have an adverse health reaction. Of the households that reported adverse reactions, 96.6% said that reactions appeared only when the nets were distributed, 0.5% at times since distribution and 2.9% all the time thereafter. Irritation of the skin was by far the most common reaction reported, occurring in 94.7% of households that reported adverse reactions. This was trailed by irritation of the eyes (45.7%) and headaches (9.6%). Very few households reported cases of nausea, vomiting or difficulty breathing. Reports of adverse reactions in the six months prior to the two-year monitoring exercise dropped to 8.3% of households and 4.3% of people surveyed. Skin irritation was reported in 100% of these households.

### Retention, coverage, and access at 24 months

Two years after distribution, 82.4% of sleeping spaces were covered with an LLIHN or an LLIN, and 86.3% of the distributed nets were retained by households. LLIN retention was higher than LLIHN retention (86.4% vs. 80.5%, z-test p < 0.001). Overall, 84.3% of individuals had access to sleeping under an LLIHN/LLIN. Additionally, households were able to cite reasons for the loss of 199 LLIHN/LLINs: 39.2% of nets were used for sleeping outside the home (in another house or outdoors), 30.2% were disposed of due to damage, 17.6% were repurposed for non-sleeping uses, 10.1% were given away as gifts, and 3.1% were stolen. Overall, 84.3% of the people had access to sleeping under an LLIHN or LLIN.

### Use

**Fig. 4:**
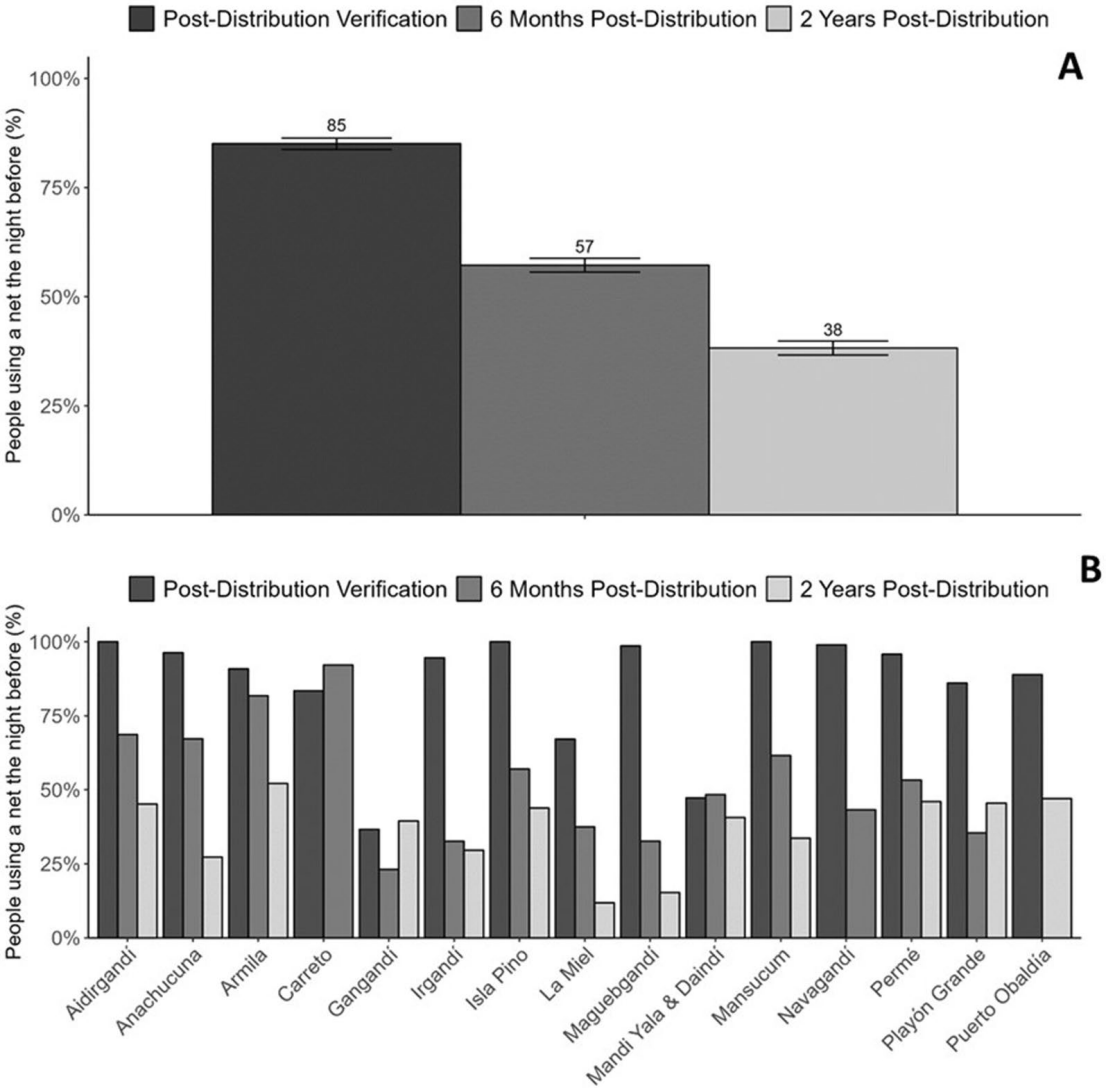
LLIHN/LLIN use across localities at verification, six months after distribution and two years after distribution globally (**A**) and by locality (**B**)

Net use decreased consistently over the monitoring periods in all localities (Fig. 4). Six months after distribution, the most common reasons for not using a net were the perceived lack of mosquitos (31.0%), the inability of the nets to keep out sandflies (16.8%), that people found it too hot to sleep under the nets (15.4%) and having insufficient nets (10.7%). Other reasons were reported less than 10% of the time (Additional file 3—Table 2). Two years after distribution, the perceived lack of mosquitoes was still the most common reason for not sleeping under a net the night before (24.3%), followed by people having either damaged (16.6%) or insufficient LLIHN/LLINs in the household (13.1%). Two years after distribution, the percentage of LLIHN/LLINs used the night before was 30.1% (differences remained, with LLIN use at 38.5% and LLIHN use at 26.0%, z-test p-value < 0.001.

### Physical integrity

**Fig. 5:**
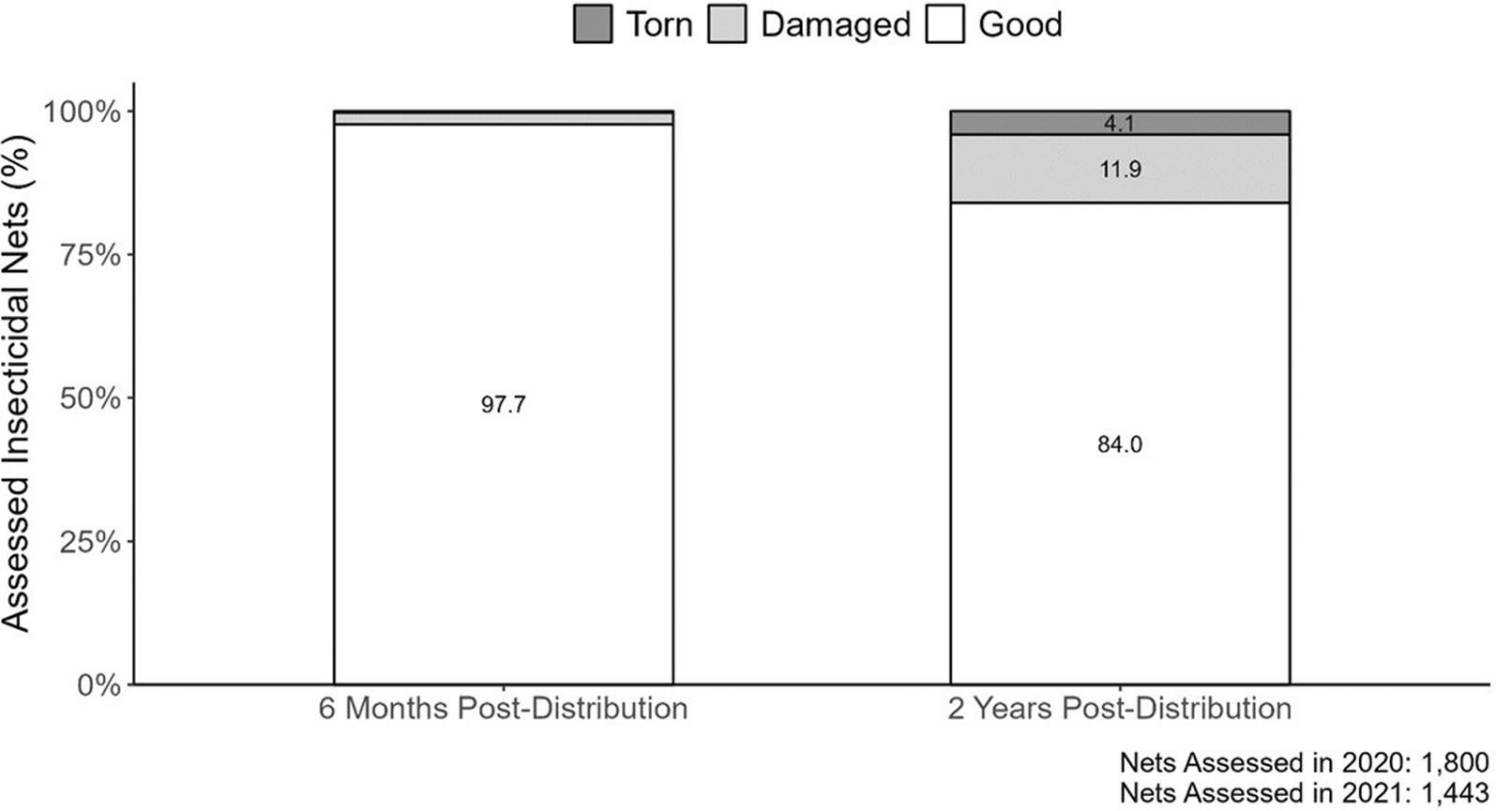
Percentage of LLIHN/LLINs in good, damaged, and torn physical conditions six months after distribution (left) and two years after distribution (right)

Six months after distribution, and across 1800 examined LLIHNs and LLINs, 12.4% of the LLINs and 4.5% of the LLIHNs presented holes. In total, 0.3% of all LLINs assessed were torn with no significant differences in the percentage of torn LLIHNs and torn LLINs (Fisher’s exact test p = 0.17) (Fig. 5). Two years after distribution, across 1443 examined LLIHNs and LLINs, holes were present in 47.3% of the LLINs and in 29.3% of the LLIHNs. In total, 4.1% of all LLIHN/LLINs evaluated were torn at this time point with significant difference between the proportion of torn LLINs and torn LLIHNs (Fisher’s exact test p = 0.003) (Fig. 5). Only 15.6% of the LLIHN/LLINs that developed holes were found to be fully repaired.

### Washing, drying, and handling practices

On average, households washed their nets 2.6 times in six months and 22.3% of them washed more than 3 times in six months. The number of washes increased between the first and second round of monitoring, (1.6 washes versus 4.0, t-test p < 0.001). Washing practices were very similar across both rounds of monitoring, with households washing their nets most commonly with bar soap (70.1%), followed by just water (21.4%), detergent (8.5%) and chlorine (0.5%). There were no differences in the use of insecticide-damaging products (chlorine and detergent) between the first and second monitoring surveys (8.7% and 9.3% respectively, z-test p = 0.74).

Most nets were dried in the shade, with only a minority of households choosing to dry their nets in the sun: 8.1% at the six-month mark, and 15.3% after two years (z-test p < 0.001). Over both monitoring surveys, 41.5% of households reportedly chose to keep them hung over their sleeping space, 37.9% raised and wrapped aside, and 14.2% fully stored away. These percentages were very similar in both monitoring rounds.

## Discussion

This pilot study aimed to evaluate the operational feasibility and acceptability of LLIHN/LLIN distributions, together with LLIHN/LLIN retention and use, before scaling up and adopting this intervention within the national integrated vector control management strategy. It demonstrates that an LLIHN/LLIN campaign is feasible and initially well received by the population in this setting, but it shows that LLIHN/LLIN use decays drastically over time, emphasizing the need for targeted SBCC efforts to sustain use.

The campaign had great acceptability in all localities, covering 97.7% of all sleeping spaces found during the distribution with very homogenous coverage across localities. The high acceptability may be linked to the fact that 59% of the households already had some kind of net before the distribution (untreated nets crafted by people using local fabric or made of mesh polyester), and to SBCC efforts made before and during the campaign that resulted in 83% of the people being aware of the distribution before it happened and 94% claiming to have received information about net’s purpose, use and handling during distribution. SBCC and community mobilization efforts may have also contributed to the low percentage of households that had to be revisited (4%). Hence, SBCC efforts should be replicated in future campaigns.

The campaign achieved high coverage of the sleeping spaces registered in 2017 (89.9% of enumerated sleeping spaces were covered). We compared this coverage with that of the sleeping spaces found during the campaign (98%). In most localities, fewer sleeping spaces were recorded during the 2019 campaign than during the 2017 enumeration and there were leftover LLIHN/LLINs after distribution. Hence, and in the absence of major known demographic changes, we suspect that some households were missed during distribution, especially in the community Maguebgandi. This highlights the importance of (1) continuing to conduct an enumeration of sleeping spaces before distribution in future campaigns so as to have a realistic denominator for the estimation of real campaign coverage, and (2) implementing a real-time monitoring system to oversee coverage during the campaign and rapidly spot underperforming localities (e.g. Maguebgandi) where to take action and bolster coverage before the campaign ends. The adoption of a digital data collection system for LLIHN/LLIN distributions will help the country execute the latter in upcoming campaigns.

Interestingly, 12% less hammocks (compared to 1% less beds) were recorded during the distribution than in the enumeration exercise of 2017. This could be due to the miscounting of daytime resting hammocks as sleeping spaces. Both the enumeration of 2017 and the distribution of 2019 aimed to only count and cover hammocks used to sleep, however, due to communication challenges posed by the local language, some hammocks may have been wrongly classified as sleeping spaces. This could explain the discrepancies in the number of sleeping hammocks counted in 2017 versus the ones counted during the campaign. Efforts should be made in future campaigns to overcome such communication barriers and accurately identify all hammocks used to sleep before and during the campaign.

The WHO recommends using a buffer of 10% when census data is older than 5 years [16]. Here, the number of procured LLIHNs and LLINs provided more than sufficient nets to achieve high coverage. This suggests that the buffer could indeed be smaller than 10% if an enumeration of sleeping spaces is conducted shortly before the campaign, as it was done in this pilot study. The cost of the pre-campaign sleeping space enumeration could be partially offset by the resulting procurement of a more accurate number of LLIHN/LLINs. This further supports the need for an enumeration of sleeping spaces short before future campaigns. In addition, assuming the default 1.8 people per net for LLIHN/LLIN quantification purposes [17] will not be an adequate strategy in this setting, as the average number of people per sleeping space was 1.34.

Despite ventilating the LLIHN/LLINs for 24 h before distribution, 38.4% of the households reported having adverse health reactions among their members. Several other studies have identified that adverse reactions can affect community perceptions towards LLIHN/LLINs and significantly reduce LLIHN/LLIN use [18–23]. Hence, such reactions may explain the rapid decay in LLIHN/LLIN use observed in this pilot. This association should be further investigated, the ventilation strategy should be revised, and ventilation time potentially increased in future campaigns.

LLIHN/LLIN retention two years after distribution was very high (86%), LLIHN/LLINs were retained in good physical condition (only 4% torn), very few were washed with aggressive products (9%) or dried under the sun (15%), and they were washed on average a bit less than once every two months (implying less than 20 washes over three years). This suggests that people retain their LLIHN/LLINs and adhere to the washing and drying recommendations provided, and that LLIHN/LLINs could preserve their bioefficacy against malaria vectors for the three-year mark (although bioefficacy was not measured to confirm this). These results contrast with findings from Nicaragua [24], Guatemala [25], the Dominican Republic (manuscript under revision) and Brazil [26], where significantly higher washing frequencies, greater use of insecticide-damaging products, much more frequent drying under the sun or higher percentages of torn nets were observed. These differences could be due to population characteristics but also to the intense SBCC and community engagement efforts implemented in Panama at the beginning of this pilot [27]. These results suggest that there is no need for top-up campaigns or alternative distribution channels in Guna Yala when campaigns achieve high coverage, but that SBCC efforts should be maintained in future campaigns.

Use decreased significantly from 85% of people sleeping under an LLIHN/LLIN six months after distribution to 38% two years after distribution despite households still having sufficient LLIHN/LLINs to cover 82% of their sleeping spaces at that time. Lack of mosquitoes was the main reason for not using the net in both surveys. However, the first survey (when high LLIHN/LLIN use was observed) was conducted during the dry season when mosquito densities were lower while the second survey was conducted when mosquito densities were higher [10]. The observed differences in use may therefore be due to overall year-round low mosquito densities in these communities. Variations in risk perceptions could be another factor affecting use, as the first survey was conducted during the high malaria transmission season and the second during the low transmission season. A relationship between LLIN use and vector abundance, risk perception and transmission seasons has been observed in other countries [28, 29] and WHO guidelines state that in areas where mosquito densities are low or where malaria transmission is low, individuals and communities may perceive less benefit to using nets [17].

Differences in retention, access, and use were observed between LLIHNs and LLINs during this pilot, suggesting a slightly lower retention and use of LLIHNs compared to LLINs. Two years post-distribution, retention of LLINs was slightly higher than that of LLIHNs (86% versus 80%) and LLIN use was higher than LLIHN use (54% of LLIN used versus 40% of LLIHN). Likely because of greater use, the percentage of torn LLINs (6%) was higher than that of LLIHNs (3%). The lower retention and use of LLIHNs could be because some hammocks are used to rest during the day and not for sleeping in these communities. LLIHNs distributed to such type of hammocks may have been left unused. This issue should be further investigated to better understand coverage and retention of LLIHNs, and whether specific actions need to be taken to improve the use of LLIHNs.

This pilot project has four main limitations. Firstly, the reasons for rejecting LLIHN/LLINs during distribution were not evaluated. Secondly, the first monitoring survey conducted six-months post-distribution did not count the total number of LLIHN/LLINs in the households, hence, attrition and overall coverage of sleeping spaces could not be calculated for this time point. Thirdly, recall bias related to the reasons for losing LLIHN/LLINs after distribution, or social desirability bias around approval of the nets or SBCC efforts may have occurred. Fourthly, bioefficacy assays could not be conducted to assess the killing effect of LLIN due to the absence of a susceptible colony of mosquito vectors in the country. Finally, LLIHN/LLIN use data was not disaggregated by sex, age, or specific population groups, yet such disaggregation is very important to identify gaps in protection as, in elimination settings, malaria tends to be concentrated in specific populations such as adult males [30, 31].

## Conclusions

LLIHN/LLINs were initially well-accepted and cared for by indigenous populations, but LLIHN/LLIN use decayed rapidly over time highlighting the need for tailored SBCC efforts to sustain use over the lifespan of the nets. The reasons for such decay in use should be further investigated to inform SBCC efforts. This pilot project generated several operationally important recommendations to be considered for the scale up of LLIHN/LLIN in Panama. Most of these recommendations have already been adopted into the country’s 2023 LLIHN/LLIN campaign.

## Supplementary Information

Supplementary Material 1.

Supplementary Material 2.

Supplementary Material 3.

## Data Availability

The data used to produce this article is available from the repository Figshare with DOI 10.6084/m9.figshare.26065087. This project contains the following files: LLIN pilot Guna Yala 2019-DATA DICTIONARY.csv. Data collection forms.pdf. Distribution_data.csv. Verification_data.csv. HH_Monitoring_data_r1_2020.csv. HH_Monitoring_data_r2_2021.csv. Net_monitoring_r1_r2.csv. The data are available under the terms of the CC BY 4.0.

## Abbreviations

DHIS2: District Health Information System-2
CHAI: Clinton Health Access Initiative
IRS: Indoor residual spraying
LLIN: Long-lasting insecticidal nets
LLIHN: Long-lasting insecticidal hammock nets
LLIHN/LLIN: refers to all nets, both LLINs and LLIHNs combined
NMCP: National Malaria Control Programme

## References

[1] Ministerio de Salud de la República de Panama. Boletines Semanales de Malaria. https://www.minsa.gob.pa/informacion-salud/malaria-1. Accessed 27 May 2024.

[2] Ministerio de Salud de la República de Panama. Plan Estratégico de Eliminación de la Malaria (PEEM) en Panama, 2018–2022. 2018.

[3] Interamerican Development Bank. Panama: Regional Malaria Elimination Intitative (RMEI) in Mesoamerica and Dominican Republic. https://www.iadb.org/en/whats-our-impact/PN-G1007. Accessed 6 Jun 2024.

[4] Organización Panamericana de la Salud. Manual para la estratificación según el riesgo de malaria y la eliminación de focos de transmissión. Washington; 2020.

[5] Magris M, Rubio-Palis Y, Alexander N, Ruiz B, Galván N, Frias D, et al. Community-randomized trial of lambdacyhalothrin-treated hammock nets for malaria control in Yanomami communities in the Amazon region of Venezuela. Trop Med Int Health. 2007;12:392–403. 17313511 10.1111/j.1365-3156.2006.01801.x

[6] WHO. World malaria report 2022. Geneva: World Health Organization; 2022.

[7] Rozendaal JA, Voorham J, Van Hoof JP, Oostburg BF. Efficacy of mosquito nets treated with permethrin in Suriname. Med Vet Entomol. 1989;3:353–65. 2519685 10.1111/j.1365-2915.1989.tb00242.x

[8] Instituto Nacional de Estadística y Censo de la República de Panama. Estimación y proyección de la población de la comarca Kuna Yala según sexo y edad. https://www.inec.gob.pa/publicaciones/Default3.aspx?ID_PUBLICACION=499&ID_CATEGORIA=3&ID_SUBCATEGORIA=10. Accessed 27 May 2024.

[9] Ministerio de Salud de la República de Panama. Analisis situacional de la region de Kuna Yala. https://www.minsa.gob.pa/sites/default/files/general/10_asisi_kuna_yala_octubre_2020_prioriza_covid_19.pdf. Accessed 23 May 2024.

[10] Ávila MI, Vajda ÉA, Gutiérrez EJ, Gibson DA, Renteria MM, Presley N, et al. *Anopheles* drivers of persisting malaria transmission in Guna Yala, Panama: an operational investigation. Malar J. 2021;20:443. 34819092 10.1186/s12936-021-03972-z PMC8611962

[11] Ministerio de Salud de Panama. Sistema WEB de vigilancia epidemiológica de Panama. https://sisvigplus.minsa.gob.pa/sisvigmalaria/. Accessed 27 May 2024.

[12] WHO. Guidelines for monitoring the durability of long-lasting insecticidal mosquito nets under operational conditions. Geneva: World Health Organization; 2011.

[13] University of Oslo. The District Health Information System (DHIS2). 2021. https://dhis2.org/downloads/archive/. Accessed 6 Jun 2024.

[14] R Core Team. R: s language and environment for statistical computing. Vienna. 2023. https://www.R-project.org/. Accessed 6 Jun 2024.

[15] Wickham H. ggplot2: elegant graphics for data analysis. New York: Springer-Verlag; 2016.

[16] WHO. Achieving and maintaining universal coverage with long-lasting insecticidal nets in malaria control. Geneva: World Health Organization; 2014.

[17] WHO. Guidelines for malaria. Geneva: World Health Organization; 2023.

[18] Iyer M, Skelton J, De Wildt G, Meza G. A qualitative study on the use of long-lasting insecticidal nets (LLINs) for the prevention of malaria in the Peruvian Amazon. Malar J. 2019;18:301. 31477112 10.1186/s12936-019-2937-1 PMC6721337

[19] Habimana A, Tuyizere M, Gikunju J, Magu D. Assessing knowledge and factors associated to long lasting insecticide nets use among pregnant women in southern Rwanda. Rwanda J Med Health Sci. 2020;3:60–70.

[20] Akello AR, Byagamy JP, Etajak S, Okadhi CS, Yeka A. Factors influencing consistent use of bed nets for the control of malaria among children under 5 years in Soroti District, North Eastern Uganda. Malar J. 2022;21:363. 36461059 10.1186/s12936-022-04396-z PMC9716664

[21] Kebede W, Tolcha A, Soboksa NE, Negassa B, Kanno GG, Aregu MB. Utilization of insecticide-treated nets in households for under-5 children and associated factors in East Mesekan District, Gurage Zone, Southern Ethiopia. Environ Health Insights. 2023;17:11786302231164287. 37007221 10.1177/11786302231164287 PMC10052613

[22] Dun-Dery F, Kuunibe N, Meissner P, Winkler V, Jahn A, Müller O. Determinants of the use of insecticide-treated mosquito nets in pregnant women: a mixed-methods study in Ghana. Int Health. 2022;14:619–31. 35064966 10.1093/inthealth/ihab087 PMC9623492

[23] Ahorlu CS, Adongo P, Koenker H, Zigirumugabe S, Sika-Bright S, Koka E, et al. Understanding the gap between access and use: a qualitative study on barriers and facilitators to insecticide-treated net use in Ghana. Malar J. 2019;18:417. 31831004 10.1186/s12936-019-3051-0 PMC6909499

[24] Villalta EL, Soto Bravo AM, Vizcaino L, Dzuris N, Delgado M, Green M, et al. Evaluation of the durability and use of long-lasting insecticidal nets in Nicaragua. Malar J. 2021;20:106. 33608024 10.1186/s12936-021-03604-6 PMC7893764

[25] Castellanos ME, Rodas S, Juárez JG, Lol JC, Chanquin S, Morales Z, et al. Evaluation of the durability of long-lasting insecticidal nets in Guatemala. Malar J. 2021;20:219. 33990197 10.1186/s12936-021-03722-1 PMC8120849

[26] da Silva Ferreira Lima AC, Galardo AKR, Müller JN, de Andrade Corrêa APS, Ribeiro KAN, Silveira GA, et al. Evaluation of long-lasting insecticidal nets (LLINs) for malaria control in an endemic area in Brazil. Parasit Vectors. 2023;16:162. 37173754 10.1186/s13071-023-05759-4 PMC10182611

[27] Ávila MI, Vajda ÉA, Jeffrey Gutiérrez E, Gibson D, Renteria MM, Presley N, et al. Entomological Surveillance Planning Tool (ESPT)-generated actionable evidence on human and vector behaviours optimizes present interventions and reduces exposure to Anopheles vectors in two communities of Guna Yala, Panamá. Malar J. 2023;22:26. 36698147 10.1186/s12936-023-04453-1 PMC9875519

[28] Koenker H, Taylor C, Burgert-Brucker CR, Thwing J, Fish T, Kilian A. Quantifying seasonal variation in insecticide-treated net use among those with access. Am J Trop Med Hyg. 2019;101:371–82. 31264562 10.4269/ajtmh.19-0249 PMC6685578

[29] Fernández Montoya L, Alafo C, Martí-Soler H, Máquina M, Malheia A, Sacoor C, et al. An evaluation of LLIN ownership, access, and use during the Magude project in southern Mozambique. PLoS ONE. 2023;18(3):e0282209. 36972236 10.1371/journal.pone.0282209 PMC10042371

[30] Cotter C, Sturrock HJW, Hsiang MS, Liu J, Phillips AA, Hwang J, et al. The changing epidemiology of malaria elimination: new strategies for new challenges. Lancet. 2013;383:900–11. 10.1016/S0140-6736(13)60310-4 PMC10583787 23594387

[31] Wen S, Harvard KE, Gueye CS, Canavati SE, Chancellor A, Ahmed BN, et al. Targeting populations at higher risk for malaria: a survey of national malaria elimination programmes in the Asia Pacific. Malar J. 2016;15:271. 27165296 10.1186/s12936-016-1319-1 PMC4863339

