# Supplementary material for: Pilot introduction of long-lasting insecticidal nets and hammock nets in the indigenous Comarca of Guna Yala, Panama
Source: Malar J. 2024 Dec 18;23:383. doi: 10.1186/s12936-024-05208-2 (PMC11657707; doi:10.1186/s12936-024-05208-2)
Supplement: Supplementary file 1 — Supplementary Material 1. [file 12936_2024_5208_MOESM1_ESM.docx]

**ADDITIONAL FILES**

**Additional file 1: Data collection forms used during the study**

**Form 1: Sleeping space enumeration**

**Form 2: LLIN distribution**

**Form 3: Verification of installation**

**Form 4: Post-distribution monitoring – round 1**

**Form 5: Post-distribution monitoring – round 2**


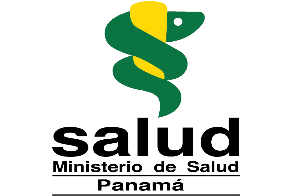
**MINISTERIO DE SALUD DE PANAMÁ**

**Formulario MTILDs #1.0**

Diario-Registro de Viviendas

**REGISTRO DE VIVIENDAS DE MOSQUITEROS IMPREGNADOS CON INSECTICIDA DE LARGA DURACIÓN (MTILDs)**

| **Fecha de Registro** | | | **Localidad** | **Corregimiento** | **Distrito** | **Región de Salud** | **Punto de Registro** | **Responsable de Registro** |
| --- | --- | --- | --- | --- | --- | --- | --- | --- |
| **Día** | **Mes** | **Año** |  |  |  |  |  |  |

| **Marca de MTILD** | **Modelo(s) de MTILD** | **Tipo de Insecticida Impregnado** | **Color de MTILD** |
| --- | --- | --- | --- |
|  | ⧠ Hamaca rectangular tamaño estándar ⧠ Cama rectangular tamaño estándar ⧠ Otro:______________ |  |  |

|  | **No. de Vivienda** | **Nombre y Apellido del Jefe de Familia** | **No. de documento de identidad** | **Número de Habitantes** | **Número de espacios para dormir**  **(MTILDs programados)** | | **Firma/huella del jefe de familia** |
| --- | --- | --- | --- | --- | --- | --- | --- |
|  |  |  |  |  | **No. de Camas** | **No. de Hamacas** |  |
| **1** |  |  |  |  |  |  |  |
| **2** |  |  |  |  |  |  |  |
| **3** |  |  |  |  |  |  |  |
| **4** |  |  |  |  |  |  |  |
| **5** |  |  |  |  |  |  |  |
| **6** |  |  |  |  |  |  |  |
| **7** |  |  |  |  |  |  |  |
| **8** |  |  |  |  |  |  |  |
| **9** |  |  |  |  |  |  |  |
| **10** |  |  |  |  |  |  |  |


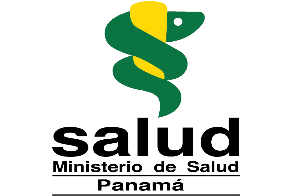
**MINISTERIO DE SALUD DE PANAMA**

**Formulario MTILDs #2.0**

Diario por Unidad Distribución/Instalación

| **Fecha** | | | **Localidad** | **Corregimiento** | **Distrito** | **Región de Salud** | **No. de unidad de distribución** | **Jefe de unidad de distribución** |
| --- | --- | --- | --- | --- | --- | --- | --- | --- |
| **Día** | **Mes** | **Año** |  |  |  |  |  |  |

**DISTRIBUCION E INSTALACIÓN DE MOSQUITEROS IMPREGNADOS CON INSECTICIDA DE LARGA DURACIÓN (MTILDs)**

|  | **No. de Vivienda** | **Nombre y Apellido del Jefe de Familia** | **No. de visita a la vivienda (X)** | | **No. de Habit.** | **No. de Espacios para Dormir** | | **No. de Mosquiteros Existentes** | | **Mosquiteros Entregados** | | **Mosquiteros Instalados** | | **¿Requiere visita de recuperación?**  **(X)** | **¿Ha escuchado los mensajes sobre los MTILDs en la radio?** | **Firma/huella de quien recibe los MTILDs** | **Observaciones** |
| --- | --- | --- | --- | --- | --- | --- | --- | --- | --- | --- | --- | --- | --- | --- | --- | --- | --- |
|  |  |  | **1^ra^** | **2^da^** |  | **Tipo Cama** | **Tipo Hamaca** | **Tipo Cama** | **Tipo Hamaca** | **Tipo**  **Cama** | **Tipo**  **Hamaca** | **Tipo**  **Cama** | **Tipo**  **Hamaca** |  |  |  |  |
| **1** |  |  |  |  |  |  |  |  |  |  |  |  |  |  | **⧠ Sí**  **⧠ No**  **⧠ N/A** |  |  |
| **2** |  |  |  |  |  |  |  |  |  |  |  |  |  |  | **⧠ Sí**  **⧠ No**  **⧠ N/A** |  |  |
| **3** |  |  |  |  |  |  |  |  |  |  |  |  |  |  | **⧠ Sí**  **⧠ No**  **⧠ N/A** |  |  |
| **4** |  |  |  |  |  |  |  |  |  |  |  |  |  |  | **⧠ Sí**  **⧠ No**  **⧠ N/A** |  |  |
| **5** |  |  |  |  |  |  |  |  |  |  |  |  |  |  | **⧠ Sí**  **⧠ No**  **⧠ N/A** |  |  |
| **6** |  |  |  |  |  |  |  |  |  |  |  |  |  |  | **⧠ Sí**  **⧠ No**  **⧠ N/A** |  |  |
| **7** |  |  |  |  |  |  |  |  |  |  |  |  |  |  | **⧠ Sí**  **⧠ No**  **⧠ N/A** |  |  |
| **8** |  |  |  |  |  |  |  |  |  |  |  |  |  |  | **⧠ Sí**  **⧠ No**  **⧠ N/A** |  |  |
| **9** |  |  |  |  |  |  |  |  |  |  |  |  |  |  | **⧠ Sí**  **⧠ No**  **⧠ N/A** |  |  |

**Observaciones:**______________________________________________________________________________________________________________________________________________________________


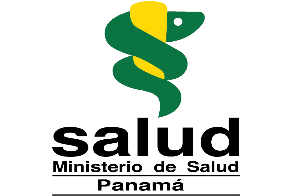
**MINISTERIO DE SALUD DE PANAMA**

**Formulario MTILDs #3.0**

Muestra de Viviendas; Supervisión/Monitoreo Rápido

| **Fecha de Supervisión/**  **Monitoreo Rápido** | | | **Localidad** | **Corregimiento** | **Distrito** | **Región de Salud** | **Supervisor Responsable de Supervisión y Monitoreo Rápido** | **Cargo del Supervisor** |
| --- | --- | --- | --- | --- | --- | --- | --- | --- |
| **Día** | **Mes** | **Año** |  |  |  |  |  |  |

**VERIFICACIÓN DE DISTRIBUCIÓN/INSTALACIÓN DE MTILDs: SUPERVISIÓN Y MONITOREO RÁPIDO**

|  | **No. de Unidad de Distr.** | **No. de Vivienda** | **Nombre y Apellido del Jefe de Familia** | **Fecha de Distribución MTILDs**  **(DD/MM/AA)** | **No. de Habit. (identificados en la visita)** | **No. de Espacios para Dormir**  **(identificados en la visita)** | | **No. de MTILDs Entregados (identificados en la visita)** | | **No. de MTILDs Instalados (identificados en la visita)** | | **¿Coinciden los datos del Form. MTILDs #2.0 con el actual?** | **No. de MTILDs Instalados correctamente** | | **¿No. de MTILDs adicionales requeridos para instalación en la vivienda?** | | **IEC sobre MTILDs** | | | | | | **¿No. de personas de la vivienda que durmieron bajo los MTILDs la noche anterior?** | **Observaciones** |
| --- | --- | --- | --- | --- | --- | --- | --- | --- | --- | --- | --- | --- | --- | --- | --- | --- | --- | --- | --- | --- | --- | --- | --- | --- |
|  |  |  |  |  |  |  |  |  |  |  |  |  |  |  |  |  | **¿Recibió mensajes IEC por parte de la unidad de distribución cuando instalaron sus MTILDs?** | **¿Sabe para qué sirven los MTILDs?** | **¿Sabe cómo lavar los MTILDs?** | **¿Sabe cómo secar los MTILDs?** | **¿Sabe cómo manejar los MTILDs cuando no están en uso?** | **¿Sabe cómo reparar los MTILDs si hay agujeros en la tela?** |  |  |
|  |  |  |  |  |  | **Tipo Cama** | **Tipo Hamaca** | **Tipo Cama** | **Tipo Hamaca** | **Tipo Cama** | **Tipo Hamaca** |  | **Tipo Cama** | **Tipo Hamaca** | **Tipo cama** | **Tipo hamaca** |  |  |  |  |  |  |  |  |
| **1** |  |  |  |  |  |  |  |  |  |  |  | **⧠ Sí**  **⧠ No** |  |  |  |  | **⧠ Sí**  **⧠ No** | **⧠ Sí**  **⧠ No** | **⧠ Sí**  **⧠ No** | **⧠ Sí**  **⧠ No** | **⧠ Sí**  **⧠ No** | **⧠ Sí**  **⧠ No** |  |  |
| **2** |  |  |  |  |  |  |  |  |  |  |  | **⧠ Sí**  **⧠ No** |  |  |  |  | **⧠ Sí**  **⧠ No** | **⧠ Sí**  **⧠ No** | **⧠ Sí**  **⧠ No** | **⧠ Sí**  **⧠ No** | **⧠ Sí**  **⧠ No** | **⧠ Sí**  **⧠ No** |  |  |
| **3** |  |  |  |  |  |  |  |  |  |  |  | **⧠ Sí**  **⧠ No** |  |  |  |  | **⧠ Sí**  **⧠ No** | **⧠ Sí**  **⧠ No** | **⧠ Sí**  **⧠ No** | **⧠ Sí**  **⧠ No** | **⧠ Sí**  **⧠ No** | **⧠ Sí**  **⧠ No** |  |  |
| **4** |  |  |  |  |  |  |  |  |  |  |  | **⧠ Sí**  **⧠ No** |  |  |  |  | **⧠ Sí**  **⧠ No** | **⧠ Sí**  **⧠ No** | **⧠ Sí**  **⧠ No** | **⧠ Sí**  **⧠ No** | **⧠ Sí**  **⧠ No** | **⧠ Sí**  **⧠ No** |  |  |
| **5** |  |  |  |  |  |  |  |  |  |  |  | **⧠ Sí**  **⧠ No** |  |  |  |  | **⧠ Sí**  **⧠ No** | **⧠ Sí**  **⧠ No** | **⧠ Sí**  **⧠ No** | **⧠ Sí**  **⧠ No** | **⧠ Sí**  **⧠ No** | **⧠ Sí**  **⧠ No** |  |  |

**Observaciones:**___________________________________________________________________________________________________________________________________________________________


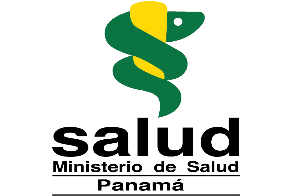
**MINISTERIO DE SALUD DE PANAMA**

**Formulario MTILDs #4.0**

Muestra de Viviendas 6 meses pos-distribución

Monitoreo de acceso, uso, mantenimiento e integridad física

| **Fecha de Monitoreo**  **Post-Distribución** | | | **Localidad** | **Corregimiento** | **Distrito** | **Región de Salud** | **Fecha de Distribución/Instalación de los MTILDs** | | | **Supervisor Responsable de Monitoreo Pos-Distribución** | | | **Cargo de Supervisor** | |
| --- | --- | --- | --- | --- | --- | --- | --- | --- | --- | --- | --- | --- | --- | --- |
| **Día** | **Mes** | **Año** |  |  |  |  | **Día** | **Mes** | **Año** |  | | |  | |
| **No. de Unidad de Distribución** | | | **No. de Vivienda** | **Nombre y Apellido del Jefe de Familia** | | **No. de Habitantes Actuales** | **No. de Espacios para Dormir actuales** | | **No. de MTILDs Entregados Anteriormente en la Campaña** | | | **No. de MTILDs Instalados Anteriormente en la Campaña** | | |
|  | | |  |  | |  | **No. de camas** | **No. de hamacas** | **Tipo**  **cama** | | **Tipo**  **hamaca** | **Tipo**  **cama** | | **Tipo**  **hamaca** |

**MONITOREO DE ACCESO, USO, MANTENIMIENTO E INTEGRIDAD FISICA DE MTILDs**

| **No. de MTILDs instalados actualmente**  **(camas o hamacas con MTILDs)** | | **No. de MTILDs instalados y utilizados**  **la noche anterior** | | **No. de personas de la vivienda que durmieron bajo los MTILDs**  **la noche anterior** | | | ***Si algún habitante de la vivienda no durmió bajo un MTILDs la noche anterior, explique la razón para no hacerlo?**  **(Indique todas razones que apliquen)** | **Marque si está conforme con:** | | | |
| --- | --- | --- | --- | --- | --- | --- | --- | --- | --- | --- | --- |
| **Tipo cama** | **Tipo hamaca** | **Tipo cama** | **Tipo hamaca** | | **Tipo cama** | **Tipo hamaca** |  | **¿El color de los MTILDs?**  **⧠ Sí ⧠ No** | **¿La tela de los MTILDs?**  **⧠ Sí ⧠ No** | **¿El tamaño de los MTILDs de cama?**  **⧠ Sí ⧠ No** | **¿El tamaño de los MTILDs de hamaca?**  **⧠ Sí ⧠ No** |

***Razón para no usar MTILDs:** 1 = No hay MTILDs suficientes; 2 = no le gusta usarlos (especifique: __________); 3 = reacciones alérgicas o molestias; 4 = no había mosquitos; 5 = hace mucho calor;

6 = MTILDs fueron robados; 7 = Se están usando afuera de la vivienda; 8 = los MTILDs fueron vendidos; 9 = por creencias; 10 = no evita la entrada de los mosquitos; 11 = Otra razón (especifique: ___________)

| **IEC sobre MTILDs**  **(Marque con X si la afirmación es cierta)** | | | | |
| --- | --- | --- | --- | --- |
| **¿Sabe para qué sirven los MTILDs?**  **⧠ Sí ⧠ No** | **¿Sabe cómo lavar los MTILDs?**  **⧠ Sí ⧠ No** | **¿Sabe cómo secar los MTILDs?**  **⧠ Sí ⧠ No** | **¿Sabe cómo manejar los MTILDs cuando no están en uso?**  **⧠ Sí ⧠ No** | **¿Sabe cómo reparar los MTILDs cuando haya huecos en la tela?**  **⧠ Sí ⧠ No** |

| **MTILD** | **Tipo de MTILD** | **Color** | **Marca** | **Forma** | **Condición del MTILD (marque lo que corresponden):** | | **No. de hoyos del MTILD** | | | |
| --- | --- | --- | --- | --- | --- | --- | --- | --- | --- | --- |
|  |  |  |  |  | **Estado de Limpieza** | **Agujeros** | **Más pequeño que un dedo**  **(0.5 -2 cm)** | **Más grande que un dedo, pero más pequeño que un puño**  **(2 - 10cm)** | **Más grande que un**  **puño, pero más pequeño que una cabeza**  **(10-25cm)** | **Más grande que una cabeza (>25cm)** |
| **No. 1** | **⧠ Cama**  **⧠ Hamaca** | **⧠Blanco**  **⧠ Azul**  **⧠ Verde**  **⧠ Otro** |  | **⧠ Rectangular**  **⧠ Circular**  **⧠ Sobre** | **⧠ Limpio**  **⧠ Sucio** | **⧠ Con agujeros/roto**  **⧠ Sin agujeros** |  |  |  |  |
| **No. 2** | **⧠ Cama**  **⧠ Hamaca** | **⧠Blanco**  **⧠ Azul**  **⧠ Verde**  **⧠ Otro** |  | **⧠ Rectangular**  **⧠ Circular**  **⧠ Sobre** | **⧠ Limpio**  **⧠ Sucio** | **⧠ Con agujeros/roto**  **⧠ Sin agujeros** |  |  |  |  |
| **No. 3** | **⧠ Cama**  **⧠ Hamaca** | **⧠Blanco**  **⧠ Azul**  **⧠ Verde**  **⧠ Otro** |  | **⧠ Rectangular**  **⧠ Circular**  **⧠ Sobre** | **⧠ Limpio**  **⧠ Sucio** | **⧠ Con agujeros/roto**  **⧠ Sin agujeros** |  |  |  |  |
| **No. 4** | **⧠ Cama**  **⧠ Hamaca** | **⧠Blanco**  **⧠ Azul**  **⧠ Verde**  **⧠ Otro** |  | **⧠ Rectangular**  **⧠ Circular**  **⧠ Sobre** | **⧠ Limpio**  **⧠ Sucio** | **⧠ Con agujeros/roto**  **⧠ Sin agujeros** |  |  |  |  |

| **No. de lavados en últimos 6 meses** | **Lavado con (marque con X):** | | | | | **Secado bajo**  **(marque con X):** | | **Manejo principal cuando los MTILDs no están en uso durante el día (marque una respuesta con X)** | | |
| --- | --- | --- | --- | --- | --- | --- | --- | --- | --- | --- |
|  | **Agua solamente** | **Jabón de barra** | **Detergente** | **Cloro** | **Otra cosa (especifique):** | **Sombra** | **Sol** | **Colgado** | **Recogido** | **Guardado** |

| **¿Algún habitante de la vivienda ha presentado reacciones secundarias o molestias luego del uso de los MTILDs?** | **Número de personas de su vivienda que han presentado reacciones secundarias o molestias luego del uso de los MTILDs** | **Tipo de reacciones secundarias o molestias (marque con X todas las que corresponden):** | | | | | |
| --- | --- | --- | --- | --- | --- | --- | --- |
| **⧠ Sí**  **⧠ No** |  | **Irritación en la piel** | **Irritación en los ojos** | **Dificultad al respirar** | **Náuseas y/o**  **vómito** | **Dolor de cabeza** | **Otro (especifique):** |

| **Fecha** | | | **Localidad** | **Corregimiento** | **Distrito** | **Región de Salud** | **Responsable** | **Cargo** | **Campaña** |
| --- | --- | --- | --- | --- | --- | --- | --- | --- | --- |
| **Día** | **Mes** | **Año** |  |  |  |  |  |  |  |

| **No. de vivienda** | **Nombre y apellido del jefe de familia** | **No. de personas** | **No. de espacios para dormir** | | **No. de MTILDs**  **instalados** | | **No. de MTILDs instalados y utilizados**  **la noche anterior** | | **No. de personas que durmieron bajo MTILDs**  **la noche anterior** | | **No. de mosquiteros convencionales (sin insecticida) instalados** |
| --- | --- | --- | --- | --- | --- | --- | --- | --- | --- | --- | --- |
|  |  |  | **Cama** | **Hamaca** | **Cama** | **Hamaca** | **Cama** | **Hamaca** | **Cama** | **Hamaca** |  |

| \| **Uso de MTILDs** \| \| \| --- \| --- \| \| ***Si algún habitante de la vivienda no durmió bajo un MTILDs la noche anterior, explique la razón para no hacerlo?**  **(Indique todas las razones que apliquen)** \| \| \| 1. No le gusta usarlos \| ⧠ \| \| 1. Reacciones alérgicas o molestias \| ⧠ \| \| 1. No hay mosquitos \| ⧠ \| \| 1. Hace mucho calor \| ⧠ \| \| 1. Se están usando fuera de la vivienda \| ⧠ \| \| 1. Por creencias \| ⧠ \| \| 1. No evita la entrada de los mosquitos \| ⧠ \| \| 1. Cambio de espacio de dormir (hamaca a cama o viceversa) \| ⧠ \| \| 1. No hay mosquiteros suficientes \| ⧠ \| \| 1. Uso de otro tipo de mosquitero (convencional) \| ⧠ \| \| 1. MTILD está roto, viejo o en mala condición \| ⧠ \| \| 1. Se siente atrapado \| ⧠ \| \| 1. Por el olor \| ⧠ \| \| 1. Durmió fuera de la vivienda la noche anterior \| ⧠ \| \| 1. Otra razón (especifique:)__________________________ \| ⧠ \| \| 1. No sabe \| ⧠ \|  \| **Posesión de MTILDs** \| \| \| \| \| --- \| --- \| --- \| --- \| \| **No. de MTILDs guardados en la vivienda**  ***(No están instalados o en uso)*** \| \| \| \| \| **Cama** \| **Hamaca** \| \| \| \| **No. de MTILDs que ya no están en la casa** \| \| \| \| \| **Cama** \| **Hamaca** \| \| \| \| **Si los MTILDs no están en la casa, explique la razón por qué no están (Indique todas las razones que apliquen)** \| \| \| \| \| 1. Se están usando fuera de la vivienda \| \| ⧠ \|  \| \| 1. Los MTILDs fueron robados \| \| ⧠ \|  \| \| 1. Los MTILDs fueron vendidos \| \| ⧠ \|  \| \| 1. Los MTILDs fueron regalados \| \| ⧠ \|  \| \| 1. Los MTILDs fueron botados por presencia de agujeros o danos en la tela \| \| ⧠ \|  \| \| 1. Uso de los MTILD para otro propósito que no sea dormir: pesca, corral, etc. \| \| ⧠ \|  \| \| 1. Otra razón (especifique: _________________________) \| \| ⧠ \|  \|  \| **Marque si está conforme con:** \| \| \| \| \| \| --- \| --- \| --- \| --- \| --- \| \| **El color de los MTILDs** \| **La textura de la tela de los MTILDs** \| **El tamaño de los MTILDs de cama** \| **El tamaño de los MTILDs de hamaca** \| **El grosor de la malla (tela) de los MTILDs** \| \| ⧠ Sí ⧠ No \| ⧠ Sí ⧠ No \| ⧠ Sí ⧠ No \| ⧠ Sí ⧠ No \| ⧠ Sí ⧠ No \| |
| --- | --- | --- | --- | --- | --- | --- | --- | --- | --- | --- | --- | --- | --- | --- | --- | --- | --- | --- | --- | --- | --- | --- | --- | --- | --- | --- | --- | --- | --- | --- | --- | --- | --- | --- | --- | --- | --- | --- | --- | --- | --- | --- | --- | --- | --- | --- | --- | --- | --- | --- | --- | --- | --- | --- | --- | --- | --- | --- | --- | --- | --- | --- | --- | --- | --- | --- | --- | --- | --- | --- | --- | --- | --- | --- | --- | --- | --- | --- | --- | --- | --- | --- | --- | --- | --- | --- | --- | --- | --- | --- | --- | --- | --- | --- | --- | --- | --- | --- | --- | --- | --- | --- | --- |

| **Mensajes promocionales sobre MTILDs** | | |
| --- | --- | --- |
| **¿Sabe para qué sirven los MTILD?** | | ⧠ Sí ⧠ No |
| **¿Sabe cómo lavar los MTILDs?** | | ⧠ Sí ⧠ No |
| **¿Sabe cómo secar los MTILDs?** | | ⧠ Sí ⧠ No |
| **¿Sabe cómo manejar los MTILDs cuando no están en uso?** | | ⧠ Sí ⧠ No |
| **¿Sabe cómo reparar los MTILDs si hay agujeros en la tela?** | | ⧠ Sí ⧠ No |
| **Manejo de MTILDs** | | |
| **No. de lavados en últimos 6 meses** |  | |
| **Lavado con:**  **(Marque con X sólo una opción)** | ⧠ Agua solamente  ⧠ Jabón de Barra  ⧠ Detergente  ⧠ Agua y jabón de barra  ⧠ Cloro  ⧠ Otro (especifique) | |
| **Secado bajo:**  **(Marque con X sólo una opción)** | ⧠ Sombra  ⧠ Sol | |
| **Manejo principal cuando los MTILDs no están en uso durante el día**  **(Marque con X sólo una opción)** | ⧠ Colgado sobre la cama o hamaca  ⧠ Recogido  ⧠ Guardado | |

| **Reacciones secundarias** | |
| --- | --- |
| **¿Algún habitante ha presentado reacciones secundarias o molestias en los últimos 6 meses de uso de los MTILDs?** | ⧠ Sí  ⧠ No |
| **No. de personas de su vivienda que han presentado reacciones secundarias o molestias en los últimos 6 meses de uso de los MTILDs?** |  |
| **Tipo de reacciones secundarias o molestias (marque con X todas las que apliquen):** | ⧠ Irritación en la piel  ⧠ irritación en los ojos  ⧠ Dificultad al respirar  ⧠ Nauseas y/o vómito  ⧠ Dolor de cabeza  ⧠ Otro (especifique)  _________________ |

| **Integridad Física de MTILDs** | | | | | | | | | |
| --- | --- | --- | --- | --- | --- | --- | --- | --- | --- |
| **Condición del MTILD**  **(Marque con una X)** | | | | **Presencia de Agujeros y Reparaciones** | | **No. de agujeros del MTILD** | | | |
| **MTILD** | **Tipo de MTILD** | **Color de MTILD** | **Modelo de MTILD (escribir el modelo que se encuentre en la etiqueta del MTILD)** | **Agujeros** | **¿Presencia de agujeros reparados?** | **Más pequeño que un dedo**  **(0.5 -2 cm)** | **Más grande que un dedo, pero más pequeño que un puño**  **(2 - 10cm)** | **Más grande que un**  **puño, pero más pequeño que una cabeza**  **(10-25cm)** | **Más grande que una cabeza (>25cm)** |
| **No. 1** | ⧠ Cama  ⧠ Hamaca | ⧠Blanco  ⧠ Azul  ⧠ Otro |  | ⧠ Con agujeros/roto  ⧠ Sin agujeros | ⧠ Sí  ⧠ No |  |  |  |  |
| **No. 2** | ⧠ Cama  ⧠ Hamaca | ⧠ Blanco  ⧠ Azul  ⧠ Otro |  | ⧠ Con agujeros/roto  ⧠ Sin agujeros | ⧠ Sí  ⧠ No |  |  |  |  |
| **No. 3** | ⧠ Cama  ⧠ Hamaca | ⧠ Blanco  ⧠ Azul  ⧠ Otro |  | ⧠ Con agujeros/roto  ⧠ Sin agujeros | ⧠ Sí  ⧠ No |  |  |  |  |
| **No. 4** | ⧠ Cama  ⧠ Hamaca | ⧠ Blanco  ⧠ Azul  ⧠ Otro |  | ⧠ Con agujeros/roto  ⧠ Sin agujeros | ⧠ Sí  ⧠ No |  |  |  |  |
| **No. 5** | ⧠ Cama  ⧠ Hamaca | ⧠ Blanco  ⧠ Azul  ⧠ Otro |  | ⧠ Con agujeros/roto  ⧠ Sin agujeros | ⧠ Sí  ⧠ No |  |  |  |  |

**Observaciones y sugerencias:**

____________________________________________________________________________________________________________________________________________________________________________________________________________________________________________________________________________________________________________________________________________________________________________________________________________________
