# Supplementary material for: Pilot introduction of long-lasting insecticidal nets and hammock nets in the indigenous Comarca of Guna Yala, Panama
Source: Malar J. 2024 Dec 18;23:383. doi: 10.1186/s12936-024-05208-2 (PMC11657707; doi:10.1186/s12936-024-05208-2)
Supplement: Supplementary file 2 — Supplementary Material 2. [file 12936_2024_5208_MOESM2_ESM.docx]

**Additional file 2:** Main indicators estimated during the pilot

**Table 1.** Main indicators estimated during the pilot

| **Indicator category** | **Indicator** | **Numerator** | **Denominator** |
| --- | --- | --- | --- |
| **LLIN/LLIHN quantification indicators** | Number of beds | N/A | N/A |
|  | Number of hammocks | N/A | N/A |
|  | People per sleeping space | Number of people in registered households | Number of sleeping spaces in registered households |
|  | People per household | Number of people in registered households | Number of registered households |
| **Pre-distribution ownership of nets** | Percentage of households that had nets (any type) before mass distribution | Number of households that had nets (any type) | Number of households registered in the areas |
| **Coverage at the end of the distribution** | Percentage of sleeping spaces that were covered with a net | Number of sleeping spaces covered with a net during distribution | Number of sleeping spaces in the localities that received nets |
| **Quality of distribution** | Percentage of sleeping spaces that were visibly covered with a net as observed during verification visits | Number of nets installed as observed during verification visits | Number of sleeping spaces found during verification visits |
|  | Percentage of nets that were properly installed above the sleeping spaces as observed during verification visits | Number of nets properly installed above sleeping spaces at the verification visits | Total number of nets installed above sleeping spaces at the verification visits |
|  | Percentage of households that required a second visit to complete distribution | Percentage of households visited a second time | Percentage of households that received nets |
| **Success of SBCC efforts** | Percentage of people who were aware of the campaign before distribution | Number of households that had heard about the campaign before it started | The total number of households that received nets |
|  | Percentage of households that claimed to know about the purpose of LLINs, their washing, drying, handling, and repairing recommendations | Number of households that claimed to know about the purpose of LLINs, their washing, drying, handling and repairing recommendations. | Total number of households that were visited for verification. |
| **Satisfaction with the distributed LLIN/LLIHN** | Percentage of people that claimed to be satisfied with the net color, fabric, and size | Number of people that claimed to be satisfied with the net color | Total number of people that were visited in the first monitoring round |
|  | Percentage of people that claimed to be satisfied with the net fabric | Number of people that claimed to be satisfied with the net fabric during the first monitoring round | Total number of people that were visited in the first monitoring round |
|  | Percentage of people that claimed to be satisfied with the size | Number of people that claimed to be satisfied with the net size during the first monitoring round | Total number of people that were visited in the first monitoring round |
| **Adverse health reaction post-distribution** | Percentage of households reporting to have adverse reactions to the LLINs among their members | Number of households reporting adverse reactions to the LLINs among their members | Number of households visited during monitoring surveys |
|  | Frequency of reaction occurrence | Number of households that reported each frequency of occurrence of the reactions | Number of households visited during monitoring surveys. |
|  | Frequency of reaction types | Number of households that reported each type of reaction | Number of households visited during the monitoring survey |
| **Coverage post-distribution** | Percentage of sleeping spaces that could be covered with a net given the nets and sleeping spaces present in the households | Number of nets present in the visited households | Number of sleeping spaces present in the visited households |
| **Retention post-distribution** | Percentage of nets that remain in the households (installed+stored) | Number of nets that were present in the visited households during monitoring rounds | Number of nets that these same households received during distribution as per distribution records |
| **Access post -distribution** | Percentage of people with access to a net | Number of people with access to a net | Number of people living in the house |
| **Use post-distribution** | Percentage of nets used the night before the survey | Number of nets used the night before the survey in visited households | Total number of nets present in the visited households |
|  | Percentage of people who used a net to sleep the night before the survey | Number of people who slept under a net the night before the survey | Total number of people in the household |
|  | Frequency of reasons for not use | Number of people that reported each reason for none-use | Total number of people that reported not using the net to sleep |
| **Physical integrity post-distribution** | Percentage of nets that had holes | Number of nets with at least one hole | Total number of nets found during monitoring surveys |
|  | Percentage of nets in torn condition (Ph> 642) | Number of nets in torn condition | Total number of nets found during monitoring surveys |
| **Washing and drying practices post-distribution** | The average number of times that households washed their nets in the last six months | Sum of times that households washed their nets in the last six months | Number of households visited during monitoring surveys |
|  | Percentage of households claiming to wash with aggressive products | Number of households that claimed washing their nets with detergent, chlorine, or other aggressive products | Total number of households visited during monitoring surveys |
|  | Frequency of products used to wash their nets | Number of households that claimed to use each type of product | Total number of households visited during monitoring surveys |
|  | Percentage of households claiming to dry their nets under the sun | Number of households that claimed to dry their nets under the sun | Total number of households visited during monitoring surveys |
