# Supplementary material for: Pilot introduction of long-lasting insecticidal nets and hammock nets in the indigenous Comarca of Guna Yala, Panama
Source: Malar J. 2024 Dec 18;23:383. doi: 10.1186/s12936-024-05208-2 (PMC11657707; doi:10.1186/s12936-024-05208-2)
Supplement: Supplementary file 3 — Supplementary Material 3. [file 12936_2024_5208_MOESM3_ESM.docx]

**Additional file 3: Additional results**

Table 1: Number of LLINs and LLIHNs installed per locality.

| **Corregimiento** | **Community** | **Targeted beds** | **Targeted hammocks** | **Distributed LLINs (bed nets)** | **Distributed LLIHNs**  **(hammock nets)** |
| --- | --- | --- | --- | --- | --- |
| **Ailigandí** | Aidirgandí | 58 | 133 | 55 | 115 |
|  | Irgandí | 36 | 287 | 38 | 273 |
|  | Playón Grande | 50 | 230 | 52 | 188 |
| **Narganá** | Gangandí | 125 | 134 | 118 | 97 |
|  | Maguebgandí | 41 | 211 | 28 | 121 |
|  | Mandi Yala & Daindí * | 80 | 129 | 118 | 123 |
| **Puerto Obaldía** | La Miel | 110 | 14 | 122 | 12 |
|  | Puerto Obaldía | 418 | 2 | 352 | 62 |
| **Tubualá** | Anachucuna | 152 | 264 | 121 | 243 |
|  | Armila | 248 | 315 | 241 | 320 |
|  | Carreto | 162 | 342 | 151 | 281 |
|  | Isla Pino | 45 | 163 | 51 | 137 |
|  | Mansucum | 42 | 426 | 45 | 393 |
|  | Navagandí | 31 | 426 | 44 | 294 |
|  | Permé | 80 | 64 | 73 | 64 |
| *Comment to explain why we’re merging two communities here. | | | | | |

Table 2. Cited reasons why household members did not sleep under an LLIBN or LLIHN the night before the survey.

| **Reason** | **Households in the first**  **Monitoring Round (2020)**  **N = 364** | | **Households in the second Monitoring Round (2021)**  **N = 374** | |
| --- | --- | --- | --- | --- |
|  | **n** | **%** | **n** | **%** |
| Allergic reactions or discomfort | 16 | 4.4 | 3 | 0.8 |
| Change in HH members affected LLIN knowledge or ownership | *Not reported* | | 14 | 3.7 |
| Changes in sleeping spaces | 13 | 3.6 | 4 | 1.1 |
| Claustrophobia or feelings of being trapped | *Not reported* | | 1 | 0.3 |
| Dislike sleeping under an LLIN | 9 | 2.5 | 22 | 5.9 |
| Excessive heat | 56 | 15.4 | 45 | 12.0 |
| House in disrepair | 10 | 2.7 | 3 | 0.8 |
| Inability to keep out sandflies | 61 | 16.8 | *Not reported* | |
| Insufficient number of LLINs in the home | 39 | 10.7 | 49 | 13.1 |
| LLINs being used outside the home | 3 | 0.8 | 36 | 9.6 |
| LLINs being washed | 26 | 7.1 | 15 | 4.0 |
| LLINs damaged or in poor condition | *Not reported* | | 68 | 18.2 |
| LLINs make a home feel cramped | 14 | 3.8 | 18 | 4.8 |
| LLINs stored away | *Not reported* | | 3 | 0.8 |
| LLINs unable to prevent mosquito entry | 2 | 0.5 | *Not reported* | |
| Perceived lack of mosquitos | 113 | 31.0 | 91 | 24.3 |
| People slept outside the home the night before | 25 | 6.9 | 3 | 0.8 |
| Preferred not to use because home received IRS | *Not reported* | | 2 | 0.5 |
| Pregnant or sick household members | *Not reported* | | 2 | 0.5 |
| Smell of LLINs | *Not reported* | | 1 | 0.3 |
| Traditional beliefs | 22 | 6.0 | 4 | 1.1 |
| Use of traditional or another type of mosquito netting (non-insecticidal) | 4 | 1.1 | 44 | 11.8 |
